# “Did I look at the blackness, or did I look at the stars?” Meaning making in stories of spirituality, mental health and recovery

**DOI:** 10.3389/fpsyg.2026.1876226

**Published:** 2026-09-03

**Authors:** Katja Milner, Paul Crawford, Alison Edgley, Mike Slade

**Affiliations:** 1 College of Health and Society, Buckinghamshire New University, High Wycombe, United Kingdom; 2 School of Health Sciences, University of Nottingham, Nottingham, United Kingdom; 3 Faculty of Nursing and Health Sciences, Nord University, Namsos, Norway

**Keywords:** meaning making, mental health, mental health and illness, narrative, recovery, religion, spirituality (MeSH), stories/storytelling

## Abstract

This qualitative narrative study explores how adults with lived experience of mental health difficulties draw on spirituality to make meaning and support recovery. Thirty participants were interviewed and asked to share their stories of spirituality, mental health and recovery. A narrative thematic analysis approach was developed to explore the superordinate theme of meaning making which comprised three interrelated themes: (1) Reframing, where spirituality enabled participants to adopt a bigger-picture perspective, evolve interpretations over time, and validate experiences; (2) Navigating mythos and logos, describing how symbolic, intuitive and experiential modes of understanding (mythos) were juxtaposed with rational, logical or scientific modes (logos) to support functional sense-making; and (3) Discerning spiritual guidance, in which intuition, synchronicity, dreams, visions and signs informed decisions, identity and coping as part of an evolving process of meaning making. Findings align with Meaning Making Framework theory, highlighting the integration of situational stressors into broader global meaning systems and the value of positive reappraisal. Mythos and logos were typically held in dynamic relationship to support coherence, agency and wellbeing, with mythos emerging as a powerful, often overlooked resource for meaning making via experiences of spiritual guidance. Drawing on findings across all three themes, the VALID (Validate, Attend, Lightly hold, Internal frameworks, Discern collaboratively) Framework for Spiritually Informed Practice is proposed. These are five evidence-derived principles offering practical orientations for engaging with spirituality at the intersection of mental health and recovery. The study offers novel, practice-relevant insights into spirituality as an intrinsic dimension of meaning-making in mental health recovery, highlighting the need to validate diverse spiritual experiences in mental health contexts.

## Introduction

*‘But meaning is something mental or spiritual. Call it fiction if you like. Nevertheless this fiction enables us to influence the course of the disease far more effectively than we could with chemical preparations.’* (Jung, 1932, p. 494)

Meaning is considered crucial in understanding human experience, especially in the context of illness and distress (Burke and Neimeyer, 2012; Park and George, 2013). According to Frankl (1992, p. 139) ‘*Once an individual’s search for meaning is successful, it… gives him the capability to cope with suffering.*’ The concepts of meaning, spirituality and mental health recovery interconnect. For example, meaning is often highlighted in definitions of spirituality and mental health recovery. However, gaps exist in understanding specific ways people utilize spiritual beliefs to make meaning from their lived experience perspectives.

While ‘spirituality’ and ‘religion’ lack universally agreed definitions, both concern symbolic systems and cultural factors which give structure and meaning to human values, behaviors and experiences (Hanegraaff, 2000; Lukoff et al., 1995). They often include a search for meaning and purpose, and connection with self, others, God or the transcendent (Gilbert, 2011; Mueller et al., 2001). Although each may have distinct aspects (e.g., public vs. private, institutional vs. individual) these are not mutually exclusive and may be expressed slightly differently by each person (Ouwehand et al., 2014). Following other contemporary researchers, spirituality and religion are viewed as overlapping concepts (Holland et al., 1998; Miller and Thoresen, 2003) with spirituality used as an broader over-arching term including both religious and non-religious expressions (Koenig et al., 2001; Royal College of Psychiatrists, 2011).

One of the most powerful worldwide developments in mental health can be viewed as the emergence of the voice of people who use mental health services (Buchanan-Barker and Barker, 2008). Their experiences have highlighted themes of resiliency, growth and transformation within the context of a deficit- and pathology-oriented mental health system (Onken et al., 2007). Recovery approaches emphasize personally-defined orientations over clinical goal-oriented understandings (Bonney and Stickley, 2008), referring to processes of discovery, healing and rebuilding meaningful life while reclaiming identity and purpose beyond mental illness effects (Anthony, 1993; Davidson and Roe, 2007; Repper, 2006).

Within lived experience accounts, spirituality emerges as a key factor, often in contrast to its general omission from mental health services (Basset and Stickley, 2010). Recent research in the field is wide ranging and includes explorations on spirituality as a tool for recovery in post-pandemic contexts (Baykal et al., 2023), recommendations around spirituality as a social determinant of health (Long et al., 2024) and spiritual influences on neuronal networks in cognitive and emotional processes in mental health treatments (Knegtering et al., 2024). Koenig and his colleagues’ (Koenig et al., 2012; Rosmarin and Koenig, 2020) meta-analysis of over 3,000 studies found substantial evidence that spiritual and religious beliefs support illness, coping and lead to positive health outcomes, including fewer depressive symptoms, lower suicide incidence, and reduced substance use severity. Positive effects of spirituality on recovery indicators include: greater empowerment and involvement in recovery enhancing activities (Yangarber-Hicks, 2004), lower suicide rates (Jarbin and von Knorring, 2004; VanderWeele et al., 2016), reduced depressive symptoms (Bosworth et al., 2003) and enhanced emotional wellness, self-esteem and life satisfaction (Baetz et al., 2002; Rosmarin and Koenig, 2020).

Some negative and harmful effects of spirituality in the context of mental health and recovery are also demonstrated. These include spiritual struggles like excessive guilt and ‘spiritual despair’ (Mohr et al., 2006). Disillusionment with faith, religious abuse scandals or anger toward God can contribute toward emotional distress and exacerbate mental health difficulties (Rosmarin et al., 2021). Stigmatizing attitudes of some religious groups in Fallot’s (2007) study regarded mental illness as a sign of moral or spiritual weakness and a systematic review found that religious and spiritual abuse and trauma can result in emotional, psychological, physical and spiritual harm (Ellis et al., 2022).

Despite the potentially powerful effects of spirituality on people’s lives, this dimension tends to be neglected in healthcare practice. A ‘religiosity’ or ‘spirituality gap’ persists, with practitioners often undervaluing spiritual factors in recovery (Dein et al., 2010) related to reported lack of knowledge, competence or practical guidance about spiritual care (McSherry and Jamieson, 2013; Mooney, 2009). Spiritual competence has been highlighted as a vital element of mental health care, enabling practitioners to better understand the role of spirituality in mental health and recovery and to consider frameworks beyond bio-medical models that help clients discover renewed meaning, motivation, and direction (Fallot, 2007; Wong-McDonald, 2007).

The connection between spirituality, mental health recovery, and meaning appears consistently across both theoretical definitions (Anthony, 1993; Gilbert, 2011) and systematic reviews of lived experience (Leamy et al., 2011; Milner et al., 2020). These studies consistently identify meaning-making as a central component of both recovery and spiritual experiences. Spiritual meaning is often communicated through narrative, as people make sense of key concerns, values, and life questions (McAdams, 1993; McTighe, 2018) fostering a sense of coherence and connection (Cook, 2016).

A distinction exists between ‘meaning in life’ and ‘meaning making.’ Meaning in life denotes purpose, significance and coherence within life experience (King et al., 2016), while ‘meaning making’ describes an active process involving the search for meaning, difficulty appraisal, and potential coping strategies during distressing or transformatory experiences (Huguelet, 2017). Spirituality can support beliefs promoting meaning making and recovery (Bassett et al., 2008; Bussema and Bussema, 2007), providing reliable meaning systems, as explored through ‘Meaning making framework’ theory (Park and George, 2013). Here, spirituality and religion are considered part of a ‘global meaning’ system which include beliefs and goals and translate into specific every day or ‘situational meaning’ which can be threatened and changed during stressful encounters (Huguelet et al., 2016). Spirituality may be particularly drawn upon during crises and mental health difficulties to make meaning when established beliefs are threatened and to weave a self-story encompassing disruption, stabilization and growth (Fallot, 1998; Gockel, 2009; Park, 2005).

Although some focus exists on spiritual meaning making in coping (Mohr et al., 2006; Park, 2005), literature lacks clear insights about such processes from lived experience perspectives in mental health and recovery contexts. Huguelet (2017) notes meaning in this context has been sparsely addressed, with most research focusing on grief rather than mental health difficulties. Yet meaning is considered crucial in understanding human experience and central to recovering from stressful and traumatic experiences (Park and George, 2013). Understanding the role of meaning making in coping with mental health challenges could inform future interventions (Huguelet, 2017).

This study is a part of the lead author’s PhD which aimed to explore the role of spirituality in mental health and recovery from lived experience perspectives to support spiritually informed mental health practice. It builds on a qualitative systematic review conducted as an initial part of this PhD which characterized empirical research around the experiences of spirituality in adults with mental health difficulties (Milner et al., 2020) and identified meaning making as a key theme.

## Methodology

This study explored the role of spirituality in mental health and recovery in the narratives of people with current or previous mental health difficulties. It derived key themes inductively, as well as investigated the superordinate theme of meaning making.

The epistemological position for this research is critical realism which assumes that a reality exists independently of our senses whilet acknowledging that knowledge is contingent and provisional (Edgley et al., 2016). Critical realism is relevant to this research topic that seeks to contribute toward mental health care approaches where the spiritual dimension is taken seriously as part of an integrated holistic approach. Within this approach where all explanations are treated as fallible (Bhaskar, 1979), service user and lived experience accounts may provide important contributions to knowledge and challenge existing assumptions.

To address the research question, a qualitative narrative research design was chosen which seeks to understand the subjective lived experiences of people and the meaning they give to these through their stories (Chase, 2011). Narrative research attempts to understand what people value and how events are connected and organized into a meaningful whole (Chase, 2011; Riley and Hawe, 2005). Stories offer a window into personal experience, drawing the listener into the teller’s point of view (Ochberg, 1988; Riessman, 1990), and provide powerful forms for expressing suffering and making sense of illness experiences (Greenhalgh and Hurwitz, 1999). Narrative research is ideally suited for accessing mental health recovery experiences and connects well with spirituality research since both relate to ideas of journey, transformation and meaning making (Ricoeur, 1984; Spector-Mersel and Knaifel, 2018).

A hermeneutic of restoration approach (Josselson, 2004; Ricoeur, 1981) provided the interpretive lens for the analysis. Characterized by a willingness to listen and to give voice to participants, this approach begins from the position that the story is being told, as best as the story-teller is able, of their subjective experience and meaning making. The researcher attempts to reveal both implicit and explicit meanings within narratives while remaining faithful to the narrator’s own intentions and perspective, presuming as little as possible (Josselson, 2004). This contrasts with a hermeneutic of suspicion, which problematises the narrative and re-authors its meaning from a position of external critical authority. This approach was not adopted here, as it does not align with the recovery-oriented and lived-experience-validating stance of this study, and risks reproducing the stigma, suspicion, and pathologisation to which spirituality and mental health experiences have historically been subject within clinical contexts.

### Methods, procedure and analysis

A Patient and Public Involvement (PPI) group provided consultation throughout the research process from people with lived experiences of mental health difficulties and spiritual beliefs, plus clinical expertise representatives (Barrett and Oborn, 2018).

Purposive sampling recruited 30 adult participants (18 + years) willing and capable of discussing their experiences of mental health difficulties and spirituality. The sample size of 30 was determined in advance, informed by narrative research norms in which sample adequacy is a matter of judgment and pragmatic considerations relative to the research question, methodology, and intended research product (Sandelowski, 1995). Participants were excluded if they currently had an in-patient admission, were using crisis services or if there was uncertainty about whether sharing their story may detrimentally impact their wellbeing. Recruitment occurred through community, mental health and religious organizations via posters and gatekeepers. Interested participants received participant information sheets before agreeing to participate. Table 1 summarizes the demographic and clinical characteristics of the participant sample.

**Table 1:** Participant characteristics.

Participant characteristics	*N* = 30 *
Mean age (years)	43
Age range (years)	20–79
Gender	
Female	17
Male	12
Non-binary	1
Ethnicity	
White British	17
White non-British	6
Asian	4
Black	1
Mixed ethnicity	1
Mental health diagnosis (*n* = 17)	
Depression	5
Anxiety	4
Bipolar disorder	3
Psychosis	2
Obsessive compulsive disorder	1
Eating disorder	1
Neurological symptoms	1
Symptoms reported (no formal diagnosis; *n* = 9)	
Depression	3
Anxiety	3
Anxiety and depression	1
Obsessive compulsive disorder	1
Post-traumatic stress disorder	1
Mental health service use	
Currently using services	11
Previously used services	12
No service use	7
Spiritual / Religious Affiliation	
Christian	7
Christian mixed with other approaches	6
Spiritual (no specific tradition)	8
Muslim	1
Hindu	1
Buddhist	1
Bahá’í	1
Pagan/Animism/Shamanism	2
Agnostic	2
Other	1

* One participant chose not to provide all demographic information.

Open-style narrative interviews were conducted in quiet, private public locations, lasting 60–90 min. Open-ended questions explored what spirituality means to participants, how they use spirituality to make sense of mental health experiences, and changes over time. Care was taken to be emotionally attentive, minimize interruptions, and create an atmosphere of trust to facilitate extended participant talk (Donovan and Sanders, 2005; Riessman, 2008).

Data analysis was based upon and adapted from narrative thematic analysis (Riessman, 2008) and Lieblich et al.’s (1998) Holistic-content narrative method. These analytical methods aim to elucidate the standpoint of the narrator and focus upon the content of what is said by participants (Riessman, 2008). The primary focus of narrative thematic analysis is on the events, experiences, content and themes within participant stories rather than how the stories are told. Narrative thematic analysis endeavors to preserve the sequence of a story and attempts to understand the themes in relation to one another as a dynamic whole, rather than fragmenting it into disparate units (Josselson, 2011).

Data analysis employed a three-stage emergent method termed ‘Narrative thematic and sequential analysis’, developed specifically for the study’s objectives, as summarized below:

Stage 1 (Preliminary) involved repeated close reading of each transcript to generate initial impressions of themes and narrative sequence. Codes were generated inductively through manual color-coding of transcripts, with marginal annotations noting salient themes, turning points, and psychospiritual processes, while holding prior theoretical frameworks loosely to avoid undue influence on the emergent analysis.

Stage 2 (Descriptive) involved systematic, in-depth engagement with each transcript individually, producing a structured ‘Story map’ document for each participant. These organized detailed thematic and sequential observations, preserving participants’ own wording and phrasing as closely as possible to remain close to their accounts. Themes were identified by their repetition, salience, and the degree of elaboration within each story, and apparent contradictions or exceptions were actively noted throughout.

Stage 3 (Integrative) moved from individual stories to cross-case analysis. Narrative summaries and an analytical mind map were created to identify patterns across the data set, in relation to superordinate themes of Meaning making, Psychospiritual development, Spiritual Connection and Characterisations of spirituality. Initial coding frameworks for each theme were then applied systematically across all thirty Story maps, with themes subsequently refined and restructured following consultation with the supervisory team. Rigor and credibility were supported throughout by an ongoing audit trail of dated methodological notes and decisions, supervisory consultation, and peer debriefing via a PPI group and specialist academic colleagues (Lincoln and Guba, 1985). Each superordinate theme generated sufficiently rich and detailed findings to warrant dedicated analysis, and accordingly the present article focuses on meaning making, with the remaining themes to be reported in subsequent publications.

Analysis was conducted by the lead researcher with regular consultations with the supervisory team. Although member checking with participants was not employed given that narrative meanings shift over time and cannot be straightforwardly validated (Riessman, 1993; Silverman, 2013), a form of respondent validation was undertaken with the PPI group in relation to preliminary findings and coding frameworks.

Although characterisations of spirituality were explored as an additional superordinate theme to illuminate how participants understood and expressed their own spiritual beliefs, participants were not systematically categorized by spiritual tradition. Consistent with the study’s narrative focus, this was a deliberate choice: meaning-making processes were understood as deeply personal and shaped by individual biography and context, and categorization by spiritual type therefore risked imposing external interpretive frameworks onto participants’ accounts.

Methodological rigor was maintained through ongoing supervision, peer debriefing, PPI consultation, audit trails, and reflexive documentation including post-interview reflections. Rigor was also enhanced by adopting a critical and reflexive approach considering researcher personal background, experience, beliefs and positionality regarding spirituality and mental health and critical examination of how the researcher’s positionality may have shaped the research process (Alvesson and Sköldberg, 2018). The lead researcher brings a longstanding personal relationship with spirituality alongside 15 years of professional experience in mental health and spiritual care. While this background provided valuable attunement to the topic and likely supported rapport with participants, it also risked unconsciously shaping recruitment, interviewing and analysis toward stories consistent with the researcher’s own framework, including over-identification with participant accounts. This is a particular concern within the hermeneutic of restoration, which requires the researcher to remain scrupulous that discovered meanings have not been substituted by their own (Josselson, 2004). Post-interview reflections documented moments where such tensions were felt in practice, supporting fidelity to participants’ own meanings throughout the analytical process. Additional reflexive strategies included a reflexive diary (Lincoln and Guba, 1985) and ongoing critical discussion of analytical decisions with the supervisory team. Peer debriefing and PPI consultation further served as opportunities to surface assumptions, receive honest feedback, and remain accountable to perspectives beyond the researcher’s own.

Ethical approval was obtained from the University of Nottingham Faculty of Medicine and Health Sciences Research Ethics Committee (Reference: 98–1809). An ‘ethical attitude’ (Josselson, 2007) was adopted recognizing that sharing personal stories about sensitive topics may increase vulnerability and distress. Interviews were conducted sensitively, prioritizing participant wellbeing, with relevant support services signposted as needed.

## Findings

The superordinate theme of meaning making is defined broadly as the ways in which participants described making sense of their experiences in relation to spirituality, mental health and recovery. After conducting the narrative analysis, three main themes were found: 1. Reframing, 2. Navigating mythos and logos, and 3. Discerning spiritual guidance (Table 2). While themes were identified systematically across all 30 participant accounts, illustrative quotes are drawn from a subset of participants on the basis of their salience and clarity in illuminating each theme, rather than to suggest that some voices were more representative than others.

**Table 2:** Meaning making coding framework.

Theme	Theme description
1. Reframing	Ways in which participants framed or constructed meaning making in relation to their spiritual beliefs and perspectives and how this functioned in relation to mental health and recovery.
1.1 Bigger picture	A form of reframing in which spirituality enabled a more effective broader perspective.
1.2 Evolving	The ways in which the process of reframing meaning was described to be fluid and evolving.
1.3 Validation	A function of reframing meaning in the context of spirituality and mental health as being validating.
2. Navigating mythos and logos	The interplay, integration and juxtaposition of spiritual ‘mythos’ frameworks with rational ‘logos’ frameworks of meaning making and their potential effects upon mental health and recovery.
3. Discerning spiritual guidance	The role of various forms of spiritual guidance (e.g., intuition, synchronicity, coincidence, signs and visions) and ways these are discerned in relation to functional meaning making, mental health and recovery.

The specific frameworks and contexts of meaning making described by participants varied according to their personal experiences and belief systems. For example, James arrived at a framework that guided his meaning and decision making through a process of exploration of spiritual information:

*“I was just looking for as much information as possible, answers about my place in the world… I came to the conclusion that there must be a God… else why would I exist… that gives me a great deal of meaning and direction…”* James

While James’s description of sense making seems quite logical, Helen talks about spiritually informed meaning making as something which more directly “makes sense” as a form of kinaesthetic knowing:

*“It [spirituality] makes sense. I mean, making sense is just, is feeling isn’t it? Sense is actually feeling the truth… finding your truth and sensing it on all levels. Yeah. But my body makes sense of things as well you know.”* Helen

The process of meaning making was not always straightforward and some participants found it confusing or difficult to articulate. Most participants’ stories were characterized by periods of questioning, doubt and uncertainty which were sometimes turning points leading to realizations or transformation. Cynthia’s narrative offers an important counterpoint, illustrating how a spiritual framework could become a source of distress rather than resolution when internal contradictions cannot be reconciled. Her account describes a previously strong faith which disintegrated into confusion and loss of meaning, with significantly negative implications for her mental health and sense of agency in recovery:

*“I find it difficult because I might have a contradiction to my faith. I think we're meant to be joyful and live life to the full and all this but I feel paralysed in a way, unable to do that… I'm stuck and can't move on in my life…I find living life at the moment with spirituality as a theme, it's very hard, to live your life when you feel as if you're contradicting everything you believe in just because of fear and not knowing what you really ought to be doing or scared to live a life on your own.”* Cynthia

Cynthia’s experience suggests that the relationship between spirituality and meaning making is not inherently beneficial and may depend upon the degree of coherence and flexibility within an individual’s spiritual framework. Where faith becomes rigid or contradictory, it may constrain rather than enable the construction of meaning. Significantly however, Cynthia’s case was notable as an exception; the majority of participants described meaning making within a spiritual context as broadly supportive of their mental health, particularly when viewed across a developmental trajectory over time. Characteristic patterns or frameworks could be discerned across participant stories which are described below.

### Theme 1: Reframing

The first theme highlights the ways in which many participants described their spiritual perspectives enabling them to reframe their experiences and ways of making meaning. This was depicted as providing various functions which supported mental health and recovery. Within his Bahá’í faith, Andrei describes reframing challenging experiences as a test with potential for positive transformation:

“*Because all I could do was… think ‘Why is this happening to me’ and try to make sense of the universe… And this is the key principle for theodicy in the Bahá’í faith. So something bad happened to you because this is taking you somewhere good if you’re willing to go with the process and have the right attitude. And it’s also a test…”* Andrei

For Andrei, a religious outlook provides balance and a fluid perspective which allows meaningful reframing that supports mental health:

*“I’ve learned of a way of thinking that makes sense… I mean that’s what religion is… you frame these things in a meaningful way… when you talk about coping with mental health and issues of any kind, this sort of perspective really really helps… After you go through this kind of terrible thing you start to see connections… you start to see the tapestry of life.”* Andrei

The process of reframing was characterized in three specific ways: as providing a ‘Bigger picture’, as ‘Evolving’ and as sometimes providing a ‘Validating’ function which are summarized in the following sub-themes below.

#### Sub-theme 1.1: bigger picture

Sometimes spiritually-informed reframing was described by participants to involve figuratively stepping back and attempting to gain a ‘bigger picture’ perspective. Lajla describes this as including her religion and relationship with God, offering comfort and better understanding of her mental health difficulties:

*“I think religion in particular helped me understand… that the problem that you're facing at the moment… It can be overcome if you just like take a step back and look at the big picture. In that sense, my anxieties disappear and so I would say in terms of recovery, it plays a great role.”* Lajla

Helen describes her spiritual framework rooted in Shamanism as providing “geography” with “tools” to navigate different internal landscapes:

*“Shamanism has been amazing really in terms of giving me a geography… tools to understand all the different landscapes that exist and how to chart one’s way through there, how to unlock things when you get stuck… it makes sense on the biggest level possible.”* Helen

Some participants used vivid metaphors which seemed to facilitate understanding. Carl utilizes the term “emotional archeology” to describe exploring his internal psychospiritual landscape:

*“So there was a necessary, emotion archaeology to be done to understand… Going back into your own past and, in the same way as you would excavate a site, looking at the features, interpreting them and saying ‘Well what did that do?’… and then you go… ‘I’m not going mad, there’s a reason for it!’ It’s reassuring in a way.”* Carl

Carl explains that spiritual practices supported management of his mental health by providing access to helpful and reframing ways of thinking:

*“… what it [spirituality] has done is given me access to ways of thinking and environments where I can both look at and deal with mental health issues… it was the fact that I went to this workshop and had this new tool, this new way of looking at things that actually helped… me to retell the story, reframe the story in a different way that sits more comfortably in my head today.”* Carl

Helen uses imagery of ‘light’ and ‘dark’ to describe reframing a dissociative traumatic experience. Her account suggests recovery allowed her to reframe “alien” blackness by understanding it as part of the landscape needed to see the stars:

*“…if I left my body and I went out into outer space… Did I look at the blackness or did I look at the stars? Which way do you face when you have nothing? Do you look at the nothing or do you look at the lights that are always there? Because there’s always light within the dark… for years I was depressed…. I was looking at the blackness…. And now I’ve made a conscious decision to look at what is there. Acknowledge the blackness, but not see it as - alien… it’s just part of the stuff of what actually is. You can’t have the stars if you don’t have the space to have the stars.”* Helen

#### Sub-theme 1.2: evolving

For some participants, reframing was described as fluid, progressive or evolving. Helen describes learning about limiting beliefs rooted in past painful experiences, and through awareness and acceptance, transforming how she reframes and relieves pain:

*“Pain tapes, so limited beliefs about myself or the world that were false and painful… if I really leapt into that pain experience and fully immersed myself into it then it changed and improved and altered. I could actually relieve the pain… You face it… And once it’s seen then it changes.”* Helen

Rachel describes the importance of allowing new meanings to evolve and be fluidly experienced rather than seeking one definitive ‘answer’:

*“I’ve had a couple of experiences where - things come up that clearly aren’t from my life… without needing to say ‘Yeah but this isn’t real because it didn’t happen,’ or… ‘I had this experience so I 100 percent believe in past lives’… what I see frequently in both myself and others is that there’s a richness of the understanding-making that a deeper meaning then comes out of.”* Rachel

Rachel suggests that openly holding evolving meanings without definitive conclusions may support psychospiritual development:

*“It’s like a lot of the beliefs I had have actually completely unravelled… there’s always an openness to the meaning then becoming something else… It feels like it’s a more organic kind of meaning making that essentially doesn’t really have an end in a way… it means that we can hold meaning with much more ease… because it is evolving and fluid…”* Rachel

Rachel’s description of meaning making as evolving and fluid suggests a way in which the reframing of meaning making may function developmentally, and to potentially support mental health and recovery.

#### Sub-theme: 1.3 validation

Some participants described how spiritual meaning frameworks enabled reframing that validated their mental health and spiritual experiences. Thomas highlights the role of Zen Buddhism in validating his challenging thought processes:

*“A lot of the Zen literature is about - thinking and how we think and difficulties in thinking… I didn’t want them to go away. I wanted them not to be a problem. And Zen seemed to be saying ‘Yeah, all of those things are completely true and thinking is a tremendously difficult thing and we have been working on how you can do it better’… They’ve got a big vocabulary for describing states of mind…”* Thomas

Thomas contrasts this with clinical approaches toward segregating illness experiences:

*“If you’re thinking about thinking about thinking… that’s an illness and we can cure that. And it felt like the Zen approach was much more ‘Oh that can happen’… And I was like ‘Yeah, this is somebody who’s not trying to divide the world into the good stuff and bad stuff’… And that with a change in focus… some of them just stopped being problems and they kind of dissolved because I understood them…”* Thomas

Rachel echoes the validating effect of not viewing mental health symptoms through a pathologising lens, but rather conceptualizing them in relation to their “essential intelligence”:

*“I think the deeper we go the more we’re able to hold the symptoms and look at them really deeply, the more they reveal their intelligence to us… And it was hugely validating… we think like ‘Oh my God, there’s something really wrong with me…’ And actually to see that all of these mechanisms started out, are rooted in the system’s own attempt to find what it needs or to heal itself in some way…”* Rachel

Noam describes how validation from his counselor regarding spirituality was supportive:

*“I’ve told the counsellor that I work with my spiritual teachers and he had to get his head around that… and he just said ‘Yes this makes sense to me’, and that was wonderful… it was helpful… it was validation.”* Noam

### Theme 2: Navigating mythos and logos

The second theme concerns how participants described and navigated two distinct frameworks of meaning making: non-rational, spiritual, mystical or symbolic ways of understanding, termed ‘mythos’, versus rational, logical or scientific frameworks, termed ‘logos’. Participants depicted the integration, juxtaposition or rejection of these forms of understanding as supporting their meaning making process and mental health recovery.

For Andrei, these contrasting frameworks support understanding through finding balance:

*“…for me it’s an issue of epistemology, it’s how you see reality and how much you learn from it. And some part of it is more objective, some part of it is more subjective and you try to correlate the two and make sure your subjective interpretation is not out of balance… for me it works, and for my health it works…”* Andrei

Carl’s story demonstrates increasing emphasis on rational logos as an important step in his recovery, providing balance to his previous emphasis on spiritual mythos:

*“I started to… let go of some of the things that… I’d been dabbling in… I’ve looked at them objectively… the whole religious dogma… I now reject. But what I have held onto is this sense of, that you can connect with the world in a more aware way… And that in itself has made me feel better…”* Carl

A significant number of participants highlighted the importance of mythos ways of understanding. Morgen, having grown up within a culture strongly emphasizing atheism and rationality, describes how spirituality provided greater self-understanding:

*“My spirituality has helped me to make sense… that I am not just a rational human being… I feel like spirituality has helped me to come to terms that it’s not all so clear-cut and it’s not all so straightforward and people are not one thing or the other, but we are all wobbling a bit about in certain spectrums…”* Morgen

Morgen explains that a spiritual shift supported her mental health and recovery by providing a counter-narrative of acceptance in relation to previously feeling unwelcome in spaces which denied spirituality:

*“I think it [spirituality] definitely had a positive impact for sure… Because I feel like a lot of… atheist discourse is incredibly racist, sexist, misogynist and homophobic…. this movement has very little to offer to me except old white men yelling at clouds…I know that when I was interacting with these spaces that my identity and who I am weren’t really acknowledged… To come into the space and be like, ‘No… we need tables and numbers and nothing else counts’, is quite an intense thing to do considering we’re just organic blobs moving about.”* Morgen

Katie explains how making sense of her mental health involved surrendering the temptation to be logical because these frameworks may not always be relevant:

*“In the mental health side of things, there's an aspect of it that involves the trust and the letting go… there has to be an element of not needing to rationally understand to overcome things as well. The objective observation helps, but the being with is the most important, because I think it's very easy to try and get logical about it all.”* Katie

Participants used various concepts to describe mythos sense making. Morgen describes the “soft” and imprecise mythos realm which includes phenomena like consciousness, imagination, dreaming and creativity:

*“…this idea of that you can’t access all of these things inside yourself and you’re… this organic blob… that people can’t a hundred percent understand or a hundred percent explain… consciousness is in itself quite spiritual with like dreaming and memorising… these are such like soft, out there kind of things we are able to do right?… imagination also… I think there is something in there that’s quite magical or quite spiritual…”* Morgen

Several participants described mystical experiences that had a profound impact. For Rachel, such experiences followed a spiritual awakening that helped her to let go of her strong inner critic:

*“So, this was probably one of the most profound, what I would now call like awakening or mystical experiences… a whole sort of series of insights… undoing of the whole kind of position of self… like, ‘unitary consciousness’ or ‘oneness’… the normal run of judgments wasn’t happening… I used to have a really strong inner critic… And it just - over the years it’s just really not there…”* Rachel

David describes mystical experiences with crystals which positively impact his mental health by helping him to feel connected:

*“I just felt a lot of loneliness, isolation… And connecting with the crystals gave me something that sort of transcended a lot of that… It makes me feel like I’m not alone for whatever reason, it connects me to something transcendent…”* David

### Theme 3: Discerning spiritual guidance

Many participants described experiences of spiritual guidance characterized by mythos phenomena such as imagination and intuition, synchronicities and coincidences, messages and signs, and visions and dreams. Some employed rational discernment to facilitate meaning making and recovery.

Noam describes intuition as an important form of spiritual guidance that helped maintain personal integrity while growing up within an oppressive culture:

*“…the vast majority of my life in fact was controlled from the outside… it was too risky to make decisions that involved how I really felt, or what I really wanted to do… But I had good intuition … knowing what to accept of the things that I was brought up on… and what to reject… So this intuition, which I consider part of the spirituality that I experienced, was there as well and very very helpful.”* Noam

Noam identifies intuition as guiding a process of healing and recovery through ‘self-therapy’, to find answers within himself.

*“I began to do something which could be defined as self-therapy. But again it’s all come from intuition, this is nothing to do with intellectual reading… it all came from within, from this part of me that was connected with that light, with that spark, intuitive… the source of my problems was within me and therefore they were transformable or solvable from within me…”* Noam

Participants commonly reported spiritual guidance through signs, synchronicities, coincidences, and visions. These manifested both as internal or mystical experiences and through external events, conversations, or interactions, and appeared across diverse spiritual belief systems. Most participants who spoke about these phenomena described positive experiences of feeling spiritually guided and supported. Charlotte shares an experience of being prayed for and receiving personally significant information:

*“Sometimes when other people are praying for you… they kind of know things that they shouldn’t know or can say things that they don’t understand but have a real significance to you… and like what would be a crazy coincidence if you didn't believe that God was involved.”* Charlotte

Elizabeth’s account of coincidence highlights the importance of a heightened sense of control, positivity and guidance:

*“I feel like I’ve got control now, of my life… I’ve got somebody guiding me… little coincidences that happen. I don’t think they are coincidences any more… Why not have these thoughts because – the more you believe in something the more power it has and the more it works. And life is just so much more positive now for me.”* Elizabeth

Other participants described potential challenges in interpretation, particularly regarding mental health difficulties. Silvia describes seeking guidance from God through coincidences while emphasizing the need for discernment: *“I’ll ask for guidance… I feel that God speaks to me through coincidences… but I have to use my brain as well… I have to weigh and test information and messages.”* She contrasts this with unhelpful messages connected to mental illness that feel “*more forceful,” “overwhelmingly true”* and may lead to *“potentially dangerous situations.”*

Alexandra similarly highlights the importance of developing discernment during recovery, describing learning to hold information critically and lightly rather than with the conviction she experiences during a psychotic episode:

*“…learning that kind of discernment… and the being unsure… whereas in psychosis that’s completely not what it is, it’s being you’re so sure that you can’t think about it. Whereas actually being able to hold something lightly and not be sure is maybe part of working out if it’s the right thing or not…”* Alexandra

Keyshia describes how embracing what she describes as a “delusion” provided guidance and greater understanding:

*“… the thing that I've come to understand is to actually embrace my delusion because it's me and it's my subconscious telling me about things about myself… it's such an intimate side of you. It's like somebody just unfolded you and told you how things are inside… actually take the delusion and follow it and create a bigger story.”* Keyshia

By accepting and giving meaning to her delusion, Keyshia created her own ending and new beginning to her recovery story:

*“I embraced my delusion as a story about me… and created the ending… And finished the delusion… I felt like I took that special part of me and I gave it a special meaning and it has an end because I came out of hospital and I've continued on with life… And I'm creating a new story.”* Keyshia

Spiritual guidance was articulated by participants to emphasize mythos frameworks which can be difficult to interpret. However, phenomena such as intuition, imagination, dreams, signs, synchronicity, coincidence and visions were described as important components of meaning making that potentially supported mental health recovery when engaged with in functional ways. Some participants described learning to navigate mythos and logos frameworks to promote discernment within challenging mental health experiences.

## Discussion

Narrative analysis findings of meaning making in the context of spirituality, mental health and recovery highlighted three main themes: Reframing, Navigating mythos and logos, and Discerning spiritual guidance. Participants demonstrated wisdom and insight in navigating complex spiritual and mental health experiences, though constructing functional frameworks was an ongoing developmental process throughout their recovery.

Participants described meaning making as a process of constructing frameworks that allowed them to reframe experiences in ways that supported their mental health and recovery. Such frameworks operated through rational (‘logos’) or spiritual (‘mythos’) understanding, though over-emphasis of either without balance or discernment could be detrimental. Spiritual perspectives often provided helpful new outlooks, validation, understanding and guidance. These findings support ‘Meaning making framework’ theory (Park and George, 2013), where meaning making involves achieving congruence between global and situational meaning. Discrepancies between these can result in distress, initiating cognitive meaning making processes to reduce this discrepancy and integrate stressors within global meaning systems, leading to better adjustment (Park, 2010). This includes deliberate coping efforts to understand situations differently through positive reinterpretations and reattributions (Folkman and Moskowitz, 2007).

Participants utilized spirituality for positive reappraisal of experiences and distress. Recent literature emphasizes how exposure to trauma, grief, and loss can elicit spiritual meaning making, which can manifest as unsettled spiritual rumination or evolve into spiritual growth and transcendence (Vis and Boynton, 2024). This narrative study extends such understanding by highlighting specific cognitive processes, notably Reframing of perspective, including bigger picture perspectives, validation of experiences, and fluid perspective changes that supported coping and recovery.

A commonly reported theme involved navigating between mythos and logos frameworks. Mythos involves personal experience, meaning, symbolism, intuition and subjective perspectives, while logos involves logic, reasoning and objectivity—both essential for human development and information processing (Zagórska, 2018), with their integration considered necessary for mature psychological functioning (Labouvie-Vief, 1994). This theme emerged directly from participants’ own descriptions of their meaning making processes across diverse spiritual traditions. They spontaneously articulated the tension and integration between these frameworks, using their own language to describe this navigation, as illustrated by Andrei’s explicit reference to correlating objective and subjective epistemologies, Katie’s articulation of surrendering the need for logical understanding, and Morgen’s distinction between the ‘soft’ imprecise realm of consciousness and imagination and more rationalist frameworks.

These categories were not experienced as a simple binary. Participant accounts consistently illustrated hybrid and fluid forms of reasoning, in which mythos and logos were held simultaneously, juxtaposed, or moved between fluidly rather than experienced as mutually exclusive. The framework is therefore perhaps better understood as describing a dynamic continuum rather than a fixed dichotomy, reflecting the complexity of lived experience. Participants narrated successfully juxtaposing these approaches for a more balanced understanding that supported their mental health and recovery. This is consistent with MISTIC study findings (Milner et al., 2020) where some participants reported effectively integrating medical (logos) language with spiritual (mythos) themes to develop ‘multicausal models of explanation’ (Ouwehand et al., 2014).

Theoretically, these findings resonate with broader scholarly arguments about the relationship between rational and non-rational ways of knowing. McGilchrist’s account suggests that left-hemisphere logos modes of attention may have come to progressively marginalize the more holistic, relational modes of understanding associated with mythos (McGilchrist and Rowson, 2013). Jung similarly argued that secular and scientific cultures have systematically devalued the interior subjective realm of the psyche, including spiritual experience, in favor of externalized, rationalist frameworks (Jung, 1964). Tacey (2013) extends this further, arguing that fear of interiority and cultural taboo around intimacy with the sacred have suppressed intrapersonal spiritual understanding, and that mythos thinking offers a pathway to reconnection with spirituality and intuition beyond logos capability. This suggests that the holistic understanding provided by mythos may be vital to health through the processes of spiritual integration:

*‘Mythos looks at the whole rather than the part, and the whole is animated by forces which the part can experience but rarely comprehend…. and our civilisation will not recover health and balance until it rediscovers a transcendental basis to its outlook.’* (Tacey, 2013, pp. 16–17)

While these theories offer broader speculative interpretive lenses that warrant further investigation, they provide useful contexts for understanding why the intrapsychic spiritual experiences described by participants may remain poorly understood or validated in contemporary clinical and cultural settings.

The theme of Discerning spiritual guidance provides novel empirical evidence highlighting intrapsychic spiritual guidance as a potentially important meaning making component. Participants described phenomena such as synchronicities, signs, messages, visions, dreams, symbols, intuition and imagination as functional components of meaning making that potentially supported mental health recovery. For participants, what mattered was not the ontological nature of these experiences, but their role in providing spiritual guidance and meaning. Recent empirical research supports the significance of synchronicity as a meaningful psychological process. Russo-Netzer and Icekson (2022) conducted a phenomenological analysis of synchronicity experiences across 45 adults. They found that the process of detecting meaning in unexpected coincidences, through receptiveness, meaningful encounter, and active meaning-detection, revalidated participants’ sense of coherence, purpose and control in life, functioning as a significant pathway to meaning. A subsequent scale validation study demonstrated that the capacity to detect meaning in synchronicity experiences predicted greater presence of meaning in life and optimism, which in turn were significant pathways to life satisfaction and wellbeing (Russo-Netzer and Icekson, 2023). These findings resonate closely with participant accounts in this study, providing empirical grounding for the role of synchronicity and potentially related intrapsychic spiritual experiences as functional components of meaning making and recovery.

Jung (1955) identified synchronicity as a frequently reported but rarely disclosed experience due to fear of ridicule, defining it as a psychic factor denoting a meaningful coincidence in time. While some have criticized Jung’s account of synchronicity (e.g., Kime, 2019), others highlight it as a function of meaning making understood through mythos frameworks (Colman, 2011). Mythos-based meaning making has also been suggested to support the understanding and navigation of psychotic experiences by introducing metaphors, symbolic themes, conflicts and resolutions that can reveal the deepest workings of the psyche and instigate transformation (Hartley, 2010; Lukoff and Everest, 1985). Jung (1921) similarly viewed imagination as key to meaning making in the context of both physical and mental illness and Tacey (2013) argues that mythos thinking supports meaning making by focusing on internal realms of the psyche, thereby promoting recovery, healing and psychospiritual growth.

Not all participants experienced spirituality as straightforwardly supportive of meaning making and recovery. Cynthia’s narrative, in which contradictions within her spiritual framework led to paralysis and loss of meaning rather than resolution, offers an important counterpoint. Her experience exemplifies what Pargament and Exline (2021) term ‘spiritual struggles’: experiences of tension, strain or conflict around spiritual matters that can become pivotal crossroads in life, leading either to greater wholeness or to psychological decline. Meta-analytic evidence from 32 longitudinal studies confirms that such struggles are prospectively linked with greater severity of mental health challenges over time (Bockrath et al., 2022). Cynthia’s experience suggests that the relationship between spirituality and meaning making is multifaceted and can be detrimental to mental health and recovery. This may depend, for example, upon the degree of coherence and flexibility within an individual’s spiritual framework. Supporting individuals to navigate such complexities therefore warrants specific clinical attention.

Participant accounts highlighted that discernment of spiritual experiences in the context of mental health difficulties was a personal developmental process, navigated from within and often through confusion and uncertainty. Alexandra distinguished the fixed, unquestionable certainty of psychotic experience from the more tentative, open quality of spiritual discernment, learning to hold experience lightly and remain open to uncertainty. Similarly, Silvia described actively weighing and testing spiritual guidance using both intuition and rational judgement, and Keyshia reclaimed her own delusional experience as a source of personal meaning and narrative agency. These findings challenge the framing of discernment as primarily a clinical task performed on or for patients, suggesting instead an intrinsic capacity that individuals develop through their own recovery journeys.

This does not imply that clinical engagement is unnecessary, but points toward a more collaborative, lived-experience validating model in which the practitioner supports individuals in developing their own spiritual sense-making, rather than adjudicating between ‘real’ spirituality and psychopathology. This might involve creating safe spaces to narrate and reflect on spiritual experiences, and attending to their functional quality, whether they expand or contract the person’s sense of agency, meaning and recovery, rather than defaulting to diagnostic categories as the primary interpretive lens. Such an approach also resonates with wider developments in contemporary meaning-centered psychotherapy. Wong’s (2020) Integrative Meaning Therapy emphasizes engagement with individuals’ existential meaning making frameworks rather than a sole focus on symptom reduction, closely mirroring the collaborative, meaning-focused approach suggested by the present findings. The clinical relevance of this broader shift is supported by emerging evidence (Marco et al., 2024), which indicates that meaning-centered psychotherapies can reduce depressive symptoms and enhance meaning in life compared to control conditions, although further methodological rigor is needed to establish their efficacy.

Drawing on findings across all three themes, five evidence-derived principles for spiritually informed practice are proposed and synthesized as the VALID Framework (see Table 3). The framework identifies Validation of the person’s spiritual framework as the foundational orientation; Attending to the function of spiritual experiences over their content; Lightly holding experiences with openness and without premature closure; supporting Internal frameworks of meaning making rather than imposing external categories; and Discerning collaboratively with the person how their spiritual framework is working for them. Derived directly from lived experience accounts, these principles offer specific, accessible orientations for practitioners, supporters and people with lived experience and are intended to complement rather than replace existing clinical assessment frameworks.

**Table 3:** The VALID framework for spiritually informed practice in mental health.

Principle	Core concept	In practice
V Validate *Validate the spiritual framework*	Authentic validation of a person’s spiritual framework is itself meaningful and supportive of recovery. Simple acknowledgement and genuine curiosity without needing to share or fully understand someone’s beliefs can be profoundly affirming. A validating response communicates that the person is the expert on their own inner life. A dismissive or pathologising response to spiritual beliefs, by contrast, may undermine the very meaning making that supports wellbeing. The starting point is openness and respect, rather than assessment.	Create space for spiritual stories Invite the person to share their spiritual beliefs and experiences. Respond with genuine curiosity rather than evaluation. Validate before assessing Aim to understand the person’s framework on its own terms before reaching for any external categories or explanations. Simple acceptance matters A straightforward affirmation that someone’s perspective makes sense even without personal agreement can be experienced as powerfully supportive.
A Attend *Attend to function over content*	What matters is not whether a spiritual experience can be explained or verified, but whether it expands or constrains the person’s sense of agency, meaning and wellbeing. Experiences such as synchronicities, signs, intuitions, visions and dreams may serve important meaning making functions regardless of how they are explained. Something that might raise concern can simultaneously be a powerful source of meaning, hope and guidance. Meaning making is an on-going process whose function may not be immediately apparent. Holding space for this unfolding meaning is key.	Attend to whether spiritual experiences are expanding or contracting the person’s sense of agency, hope and meaning. Ask how the experience functions in the person’s life and what it means to them, before reaching for any explanatory framework. An experience can be both spiritually meaningful and worth attending to carefully. These are not mutually exclusive.
L Lightly Hold *Support openness and uncertainty*	The capacity to hold spiritual experiences lightly with curiosity and without needing definitive answers may itself be a sign of healthy meaning making. Meaning making is often a fluid, evolving process rather than something that arrives at fixed conclusions. Remaining in productive uncertainty and allowing meanings to shift over time, can be more valuable than reaching for premature certainty. The ability to hold experiences with openness and flexibility may be distinguishable from the fixed, unquestionable certainty that can characterize more distressing states, making this a meaningful indicator in its own right.	Model open exploration Adopt a stance of curious enquiry rather than definitive conclusion. Invite exploration rather than explanation. Support productive uncertainty Validate doubt, questioning and evolving meanings as a healthy part of the meaning making journey rather than signs of confusion. Notice how things are held Attend to whether experiences are held with flexibility and openness, or with fixed, unquestionable conviction.
I Internal Frameworks *Scaffold the person’s own meaning making*	People develop their own sophisticated internal frameworks for making sense of spiritual experiences alongside mental health, drawing on both intuitive and rational ways of knowing. Rather than applying external judgements about which experiences are valid or concerning, the task may be to support the person’s own developing internal frameworks — accompanying rather than directing their sense-making. This involves supporting both intuitive, symbolic and spiritual ways of knowing alongside rational frameworks, helping the person draw upon and consider both where relevant to them.	Ask how the person themselves makes sense of their experiences. Support their own frameworks rather than offering external conclusions. Support diverse ways of meaning making Where relevant, invite reflection upon different ways of making meaning including spiritual/intuitive and rational ways as they each may have different perspectives to teach. Ask rather than tell ‘How does this sit with you?’ and ‘What does this mean to you?’ are more powerful starting points than offering interpretations.
D Discern Collaboratively *Explore flexibility and coherence together*	Spiritual frameworks may not be inherently beneficial — where they become rigid or contradictory, they may constrain rather than support meaning making and wellbeing. This may be best explored collaboratively as a shared enquiry with curiosity and openness. Periods of doubt and uncertainty are not necessarily signs of difficulty; they may frequently be turning points toward growth and transformation. A collaborative stance allows these periods to be navigated together with genuine curiosity rather than alarm, honoring the person’s own evolving capacity for discernment.	Explore together with curiosity Invite reflection on how the person’s spiritual framework is currently working for them — whether it feels alive and evolving, or stuck and limiting. Sit with contradiction Where doubts or contradictions emerge, resist resolving them prematurely. Uncertainty within a spiritual framework may be a sign of growth. Together, attend to whether the framework expands or limits the person’s capacity to engage with life, find meaning and sustain hope.

A practice framework derived from lived experience accounts of spirituality, meaning-making and mental health recovery.

These principles are offered as reflective orientations for anyone engaging with the intersection of spirituality and mental health — whether as a practitioner, trainer, researcher, or person with lived experience. They are derived directly from the wisdom of those who have navigated these experiences themselves.

Ideally, the summary given here:

Validate: validate the spiritual framework.

Attend: attend to function over content.

Lightly hold, support openness and uncertainty.

Internal frameworks, scaffold the person’s own meaning making.

Discern collaboratively, explore flexibility and coherence together.

The VALID framework is not intended as a prescriptive protocol but as a set of reflective orientations derived from lived experience accounts. The principles are intended to complement rather than replace existing clinical assessment and risk frameworks, and are offered as a recovery-oriented commitment to lived experience expertise and collaborative, person-centered practice.

Underpinning both the findings and the VALID framework is the hermeneutic of restoration adopted in this study, which foregrounds participants’ own constructions of meaning, rather than adjudicating their validity against clinical categories of pathology or risk. This reflects a deliberate choice to privilege lived experience accounts historically marginalized within clinical framings of spirituality and mental health, though a hermeneutic of suspicion might illuminate further dimensions, and future research adopting a more critical interpretive stance could usefully complement these findings.

### Study limitations and future research

While narrative methodology supported the collection of rich, in-depth data, it can be criticized as less systematic than other approaches, and findings are necessarily interpretive, tentative, and context-dependent, reflecting the specific stories participants chose to share at that time (Riessman, 2008). Although narrative thematic analysis suited the study’s objectives, it risks overgeneralising across stories and obscuring individual meaning-in-context, and any thematic categorization may inadequately capture the complexity of whole-story narratives (Frank, 2010; Riessman, 2008). Furthermore, the psychospiritual focus of this research meant that social and cultural factors were not emphasized to the same degree, which may have limited the illumination of contextual and co-constructed aspects of participants’ narratives. While participant narratives captured retrospective accounts of psychospiritual development over time, and analytical attention was paid to the sequential and temporal dimensions of stories, the cross-sectional nature of data collection means the study cannot account for how meaning making processes may continue to evolve beyond the point of interview. Prospective longitudinal research would offer valuable complementary insight into the ongoing temporal dynamics of psychospiritual meaning making across recovery trajectories.

A further limitation concerns the likelihood of self-selection bias. Recruitment through religious, community, and mental health organizations may have preferentially attracted individuals with a positive or well-developed relationship with spirituality. Those for whom spirituality was experienced as harmful or distressing, including individuals with experiences of religious trauma or spiritual abuse may have been underrepresented (Ellis et al., 2022; Rosmarin et al., 2021). Those currently experiencing more acute or unresolved mental health difficulties were also excluded by design, which may have further shaped the overall trajectory of stories collected. While the sample had some diversity in terms of spiritual tradition and ethnic background, the study was conducted within a UK context, and the cultural specificity of this setting may shape findings in ways that limit transferability to other cultural and national contexts.

Taken together, these factors mean caution should be exercised regarding the transferability of findings. Future research would benefit from more targeted recruitment strategies designed to capture a fuller diversity of spiritual experiences, including those marked by conflict, ambivalence, or harm and from samples drawn from a wider range of cultural and national contexts. Beyond addressing these limitations, the novel findings of this study also point toward several productive future directions. The theme of Discerning spiritual guidance, identified here as a potentially important but underexplored dimension of meaning making, warrants further dedicated investigation. The intrapsychic spiritual guidance experiences described by participants, including synchronicities, intuition, dreams and signs, and the processes by which individuals develop discernment in relation to these, represent a rich area for future qualitative and longitudinal research. The VALID Framework for Spiritually Informed Practice, derived from these findings, also invites empirical evaluation; testing its utility and acceptability in clinical practice settings, and exploring whether practitioner training grounded in its principles supports effective engagement with spirituality in mental health contexts.

## Conclusion

This study demonstrates that spirituality can play a central role in meaning making for people living with mental health difficulties, operating through reframing, the dynamic integration of mythos and logos, and the discerning use of spiritual guidance. Participants’ narratives showed how bigger-picture perspectives, validation of experience, and openness to evolving meanings can reduce distress and support recovery. Mythos-based processes such as intuition, symbolism and synchronicity were not ends in themselves but resources for meaning making, often most generative when met with reflective discernment and held in dynamic relationship with logos. Where this interplay became rigid or one mode dominated at the expense of the other, confusion or distress could follow. Mythos, however, emerged as a potentially powerful yet overlooked contributor to intrapsychic meaning making and spiritual guidance.

These findings offer specific, data-grounded practice implications, synthesized here as the VALID Framework for Spiritually Informed Practice (see Table 3). Derived directly from lived experience accounts, VALID offers five reflective orientations — Validate, Attend, Lightly hold, Internal frameworks, Discern collaboratively — designed to support people engaging with spirituality at the intersection of mental health and recovery. The framework is intended to complement rather than replace existing clinical frameworks, and represents a meaningful step toward translating qualitative findings into accessible, practice-relevant guidance.

Spirituality represents a potent, multifaceted and still insufficiently understood dimension of meaning making in mental health recovery that deserves sustained research attention and greater integration into clinical practice. Future research building on these findings could deepen understanding of psychospiritual meaning making across diverse contexts, further develop and evaluate the VALID Framework, and explore the underexamined dynamics of intrapsychic spiritual guidance and discernment. This study offers a grounded contribution to spiritually informed mental health care rooted in lived experience that places meaning making at the heart of recovery, as something to be understood, supported, and validated.

## Data Availability

The datasets presented in this article are not readily available because this is confidential and potentially sensitive information of detailed participant stories. Any inquiries about the datasets should be directed to the corresponding author.
